# Interaction of smoking and obesity susceptibility loci on adolescent BMI: The National Longitudinal Study of Adolescent to Adult Health

**DOI:** 10.1186/s12863-015-0289-6

**Published:** 2015-11-04

**Authors:** Kristin L. Young, Misa Graff, Kari E. North, Andrea S. Richardson, Karen L. Mohlke, Leslie A. Lange, Ethan M. Lange, Kathleen M. Harris, Penny Gordon-Larsen

**Affiliations:** Department of Epidemiology, Gillings School of Global Public Health, University of North Carolina, Chapel Hill, NC USA; Carolina Population Center, Gillings School of Global Public Health, University of North Carolina, Chapel Hill, NC USA; Carolina Center for Genome Sciences, Gillings School of Global Public Health, University of North Carolina, Chapel Hill, NC USA; Department of Genetics, Gillings School of Global Public Health, University of North Carolina, Chapel Hill, NC USA; Department of Sociology, Gillings School of Global Public Health, University of North Carolina, Chapel Hill, NC USA; Department of Nutrition, Gillings School of Global Public Health, University of North Carolina, Chapel Hill, NC USA; 137 East Franklin Street, Suite 306, Chapel Hill, NC 27514 USA

**Keywords:** Adolescence, Obesity, Smoking, Gene-environment interaction

## Abstract

**Background:**

Adolescence is a sensitive period for weight gain and risky health behaviors, such as smoking. Genome-wide association studies (GWAS) have identified loci contributing to adult body mass index (BMI). Evidence suggests that many of these loci have a larger influence on adolescent BMI. However, few studies have examined interactions between smoking and obesity susceptibility loci on BMI. This study investigates the interaction of current smoking and established BMI SNPs on adolescent BMI. Using data from the National Longitudinal Study of Adolescent to Adult Health, a nationally-representative, prospective cohort of the US school-based population in grades 7 to 12 (12–20 years of age) in 1994–95 who have been followed into adulthood (Wave II 1996; ages 12–21, Wave III; ages 18–27), we assessed (in 2014) interactions of 40 BMI-related SNPs and smoking status with percent of the CDC/NCHS 2000 median BMI (%MBMI) in European Americans (*n* = 5075), African Americans (*n* = 1744) and Hispanic Americans (*n* = 1294).

**Results:**

Two SNPs showed nominal significance for interaction (*p* < 0.05) between smoking and genotype with %MBMI in European Americans (EA) (rs2112347 (*POC5*): β = 1.98 (0.06, 3.90), *p* = 0.04 and near rs571312 (*MC4R*)*:* β 2.15 (−0.03, 4.33) *p* = 0.05); and one SNP showed a significant interaction effect after stringent correction for multiple testing in Hispanic Americans (HA) (rs1514175 (*TNNI3K*): β 8.46 (4.32, 12.60), *p* = 5.9E-05). Stratifying by sex, these interactions suggest a stronger effect in female smokers.

**Conclusions:**

Our study highlights potentially important sex differences in obesity risk by smoking status in adolescents, with those who may be most likely to initiate smoking (i.e., adolescent females), being at greatest risk for exacerbating genetic obesity susceptibility.

**Electronic supplementary material:**

The online version of this article (doi:10.1186/s12863-015-0289-6) contains supplementary material, which is available to authorized users.

## Background

Adolescence is a sensitive period for weight gain and health risk behaviors, such as smoking [1, 2]. Obese smokers suffer 2.8–3.7 times greater mortality than those who are not obese and do not smoke [3]. In the US, nearly 90 % of adult daily smokers begin smoking in their teens [4], and 400,000 adolescents become daily smokers every year [5]. Many adolescents, particularly females, use smoking as an appetite control strategy [6, 7]. Females with greater body dissatisfaction are more likely to smoke [8], and obesity increases the likelihood of being highly addicted to nicotine during adolescence [9]. The effects of smoking differ by gender, in that smoking has a reported antiestrogenic effect in females, which may influence fat deposition [10, 11]. Adolescent smoking also varies by ethnicity, with Hispanic teens that have expressed concern about their weight being more likely to smoke than non-Hispanic teens [12]. While it has been demonstrated that weight is generally lower among adult smokers (ages 25–44 years), and higher among former adult smokers, this trend has not been observed in some younger smokers (ages 16–24 years) [13]. In addition, weight control effects of smoking may dissipate over time, as long-term smokers (20+ years) are heavier than never or former smokers, and heavy smokers are more likely to be obese than both other smokers and nonsmokers [14, 15].

Genome-wide association studies (GWAS) have identified single nucleotide polymorphisms (SNPs) contributing to variation in adult body mass index (BMI) [16–21], and evidence suggests these loci may have the greatest influence on adolescent BMI [22–28]. While many studies of obesity control for smoking status [29–32], few have examined the interaction between smoking and obesity susceptibility loci on BMI [33–36]. However, smoking has been implicated in appetite suppression through the *POMC* neural pathway [37], and loci in this pathway *(POMC* and *MC4R)* increase obesity risk [18, 38]. Our study examines the interaction between current smoking and 40 GWAS-identified and replicated SNPs associated with BMI in European descent adults [16, 18, 19, 21] on adolescent BMI in a multiethnic nationally-representative cohort.

## Results

Sample size, gender, mean age, percent median BMI (%MBMI), smoking status and other descriptives are presented by ancestry in Table 1. In the full sample, 11 % of participants aged 12–21 were obese (BMI ≥ 95th percentile), while a further 17 % were overweight (BMI ≥ 85^th^ percentile). African Americans (AA) had the highest percent obese (15.8 %), while Hispanic Americans (HA) had the highest percent overweight (21.9 %). Two-sample t-tests showed significantly higher BMI and %MBMI in female, but not male, smokers than their non-smoking counterparts (Additional file 1: Table S1).

**Table 1:** Sex, age, BMI, %MBMI and smoking status by ethnicity in the Add Health analytic sample

Characteristic	All (*N* = 8113)	European Americans (*N* = 5075)		African Americans (*N* = 1744)		Hispanic Americans (*N* = 1294)	
	Mean [95 % CI] /*N* (%)	Smokers (*N* = 2065)	Nonsmokers (*N* = 3010)	Smokers (*N* = 324)	Nonsmokers (*N* = 1420)	Smokers (*N* = 367)	Nonsmokers (*N* = 927)
Female	4286 (52.8)	1102 (53.4)	1569 (52.1)	149 (46.0)	811 (57.1)	183 (49.9)	472 (50.9)
Age in years	16.36 [16.32,16.40]	16.60 [16.53, 16.68]	16.08 [16.02, 16.15]	16.75 [16.54, 16.95]	16.34 [16.24, 16.43]	16.66 [16.48, 16.84]	16.53 [16.41, 16.64]
BMI	23.45 [23.34, 23.57]	23.18 [22.96, 23.40]	22.94 [22.78, 23.12]	24.97 [24.31, 25.63]	24.13 [23.83, 24.43]	23.65 [24.12, 25.27]	24.70 [23.32, 23.99]
%MBMI	112.42 [111.88, 112.96]	110.40 [109.36, 111.45]	110.76 [109.93, 111.59]	118.52 [115.36, 121.67]	115.93 [114.49, 117.36]	112.83 [114.64, 120.06]	117.35 [111.25, 114.41]
Self-reported BMI	79 (0.01)	24 (0.01)	30 (0.01)	6 (0.02)	12 (0.01)	2 (0.005)	5 (0.005)
% Obese	11 %	11 %	9 %	18 %	14 %	17 %	11 %
% Overweight	17 %	17 %	16 %	19 %	20 %	22 %	19 %
Region of US							
West	1546 (19.1)	247 (12.0)	533 (17.7)	38 (11.7)	208 (14.6)	146 (39.8)	347 (40.3)
Midwest	2286 (28.2)	824 (39.9)	1034 (34.4)	65 (20.1)	268 (18.9)	39 (10.6)	56 (6.0)
South	3234 (39.8)	686 (33.2)	987 (32.8)	200 (61.7)	866 (61.0)	108 (29.4)	387 (41.8)
Northeast	1047 (12.9)	308 (14.9)	456 (15.1)	21 (6.5)	78 (5.50)	74 (20.2)	110 (11.9)
African Americans							
Highly Educated				49 (15.1)	291 (20.5)		
Hispanic Americans							
Ancestry							
Puerto Rican						89 (24.3)	134 (14.5)
Cuban						37 (10.0)	156 (16.8)
Mexican						181 (49.3)	475 (51.3)
Central/South American						27 (7.4)	92 (9.9)
Other Hispanic						33 (9.0)	70 (7.5)
Immigrant status							
US Born						325 (88.6)	702 (75.7)
Non-US born						42 (11.4)	225 (24.3)

In main effects analyses of SNPs on %MBMI among European Americans (EA), 33 of the established 39 BMI SNPs were directionally consistent with previous results [18], and 19 of those showed nominally significant association with %MBMI (Additional file 2: Table S2). In AA, 12 out of 17 generalizable SNPs had effects on %MBMI that were directionally consistent with the published literature, and 5 of these were nominally associated with %MBMI (Additional file 3: Table S3). In our HA sample, 22 out of 31 established BMI loci in HA were directionally consistent with effects reported for BMI in EA adults, and 3 of these were nominally associated with %MBMI (Additional file 4: Table S4). Interaction analyses were subsequently performed for these 33, 12 and 22 directionally consistent SNPs in EA, AA and HA, respectively.

**Fig. 1:**
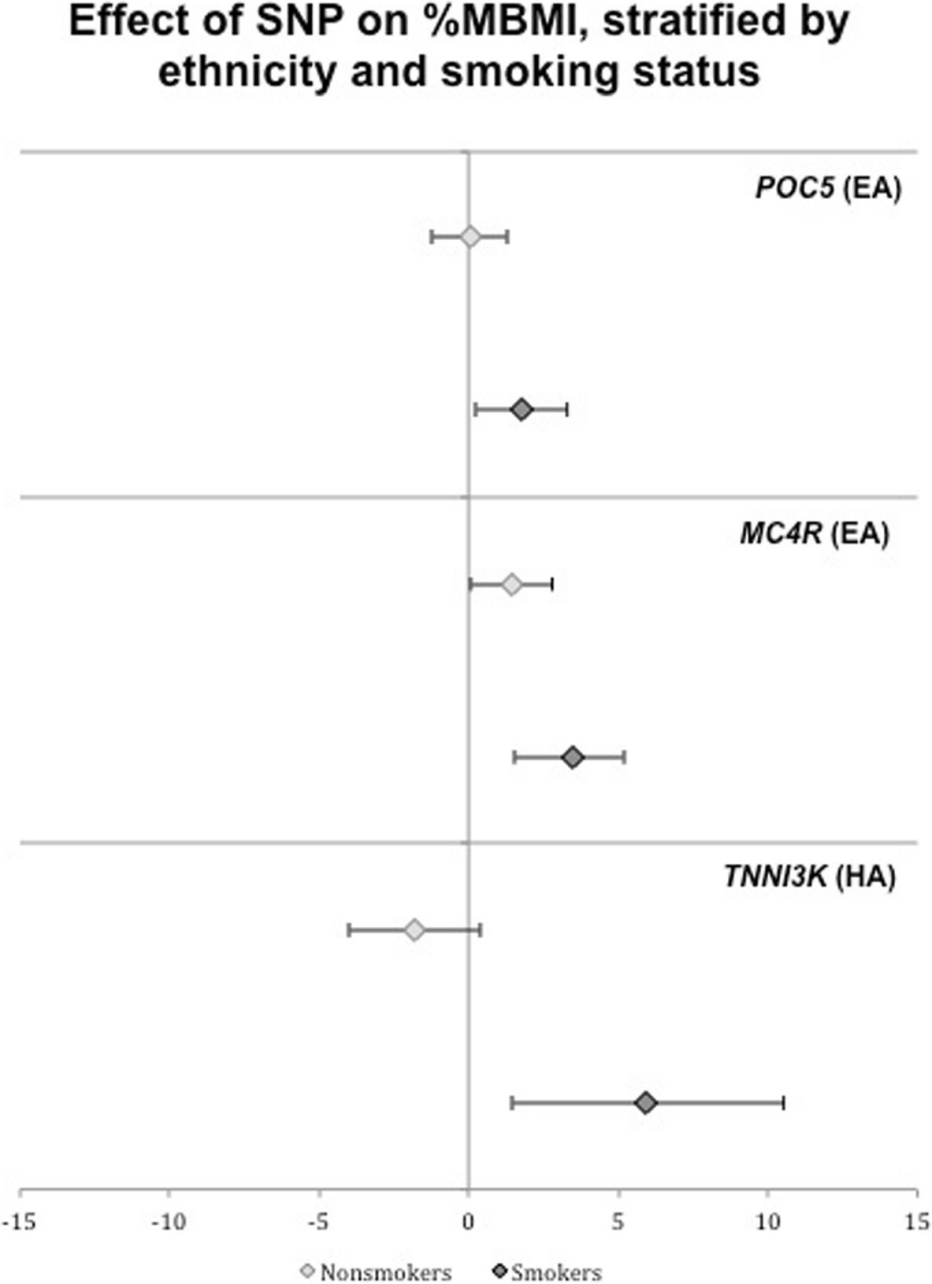
Main effect of SNP on %MBMI, stratified by ethnicity and smoking status, for those SNPs which showed a nominally significant (p<0.05) interaction effect with smoking on %MBMI

Two SNPs showed nominal (*p* < 0.05) evidence for interaction with smoking on %MBMI in EA adolescents [rs2112347 *(POC5)*: β = 1.98 (0.06, 3.90), *p* = 0.04 and near rs571312 *(MC4R):* β 2.15 (−0.03, 4.33) *p* = 0.05]. One SNP had a significant interaction effect after the most stringent multiple test correction for 67 SNPs tested across three ancestries (0.05/67 = 7.5E-04) in HA adolescents [rs1514175 *(TNNI3K)*: β = 8.46 (4.32, 12.60), *p* = 5.9E–05] (Additional file 2: Tables S2, Additional file 3: Tables S3 and Additional file 4: Tables S4). Fig. 1 illustrates results from stratified analyses of these SNPs on %MBMI by smoking status. In all cases, the estimated effect of the BMI-increasing allele was more pronounced in smokers (Fig. 1 and Table 2). None of these SNPs showed a main effect on smoking status (Additional file 2: Table S2, and Additional file 4: Table S4).

**Table 2:** Stratified analysis of nominally significant (*p* < 0.05) SNP-by-smoking interactions on %MBI in Add Health

	European American (EA) Nonsmokers		European American (EA) Smokers	
*POC5* (rs2112347)	Beta [95 % CI]	*p*	Beta [95 % CI]	*p*
All	0.04 [−1.19, 1.27]	0.947	1.75 [0.22, 3.28]	**0.025**
Females	1.22 [−0.49, 2.93]	0.16	2.76 [0.55, 4.97]	**0.014**
Males	−1.48 [−3.26, 0.30]	0.102	1.03 [−1.01, 3.07]	0.324
*MC4R* (rs571312)	Beta [95 % CI]	*p*	Beta [95 % CI]	*p*
All	1.46 [0.09, 2.83]	**0.036**	3.50 [1.78, 5.22]	**7.54E–05**
Females	1.11 [−0.79, 3.01]	0.253	5.48 [3.07, 7.89]	**8.37E–06**
Males	2.05 [0.07, 4.03]	**0.042**	0.87 [−1.58, 3.32]	0.489
	Hispanic American (HA) Nonsmokers		Hispanic American (HA) Smokers	
*TNNI3K* (rs1514175)	Beta [95 % CI]	*p*	Beta [95 % CI]	*p*
All	−1.80 [−4.09, 0.49]	0.123	5.97 [2.36, 9.58]	**0.001**
Females	−2.00 [−5.21, 1.21]	0.223	6.41 [0.92, 11.90]	**0.022**
Males	−1.78 [−4.99, 1.43]	0.279	5.25 [0.39, 10.11]	**0.033**

Bold highlights nominally significant associations (*p* ≤ 0.05). Mixed effects model, BMI = β **+** βSNPxSMK + βSNP + βSMK + βage + βsex + f + s + **ε**, Betas shown in table refer to βSNPxSMK. %MBMI = Percent of the CDC/NCHS 2000 median BMI

**Fig. 2:**
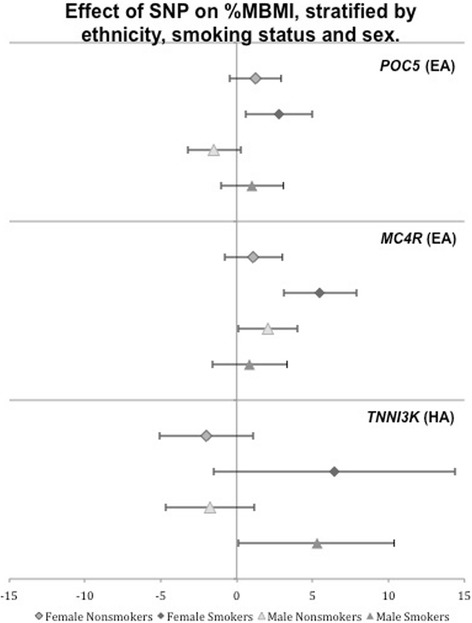
Main effect of SNP on %MBMI, stratified by ethnicity, smoking status, and sex, for those SNPs which showed a nominally significant (p<0.05) interaction effect with smoking on %MBMI

Examination of three-way interactions (SNP x smoking status × sex) for these three SNPs revealed only *MC4R* had a nominally significant interaction effect [β = 5.44 (1.11, 9.77), *p* = 0.014]. Given the available sample sizes, it is not unexpected that statistical evidence supporting a three-way interaction would be difficult to detect. When we investigated SNP × smoking status interaction for *MC4R* in EA stratified by sex, we found a nominally significant interaction only in EA females [β = 4.75 (1.73, 7.77), *p* = 2.0E-03; EA males β = 1.09 (−4.23, 2.05), *p* = 0.50]. In addition, when we stratified the effect of the obesity-risk genotype by sex and smoking status, we noted differential association with %MBMI (Table 2). None of the three loci that showed nominal significance for interaction were associated (*p* < 0.05) with %MBMI in female nonsmokers, while only *MC4R* was nominally significant in male nonsmokers. Both *TNNI3K* [β = 6.41 (0.92, 11.90), *p* = 0.02] and *POC5* [β = 2.76 (0.55, 4.97), *p* = 0.01] were nominally significant in HA and EA female smokers, respectively. *MC4R* was significant after correction for multiple testing in EA female smokers [β = 5.48 (3.06, 7.88), *p* = 8.4E-06] (Fig. 2).

## Discussion

While previous research has shown that some smoking-associated loci influence BMI in smokers but not never smokers [39], and some established BMI loci are associated with smoking [40], few studies have examined the interaction between smoking and genetic risk for obesity on adolescent BMI. In this nationally representative study of adolescents, we identify two nominally significant obesity susceptibility variants in EA, rs2112347 (*POC5*) and rs571312 (*MC4R*), and one Bonferroni corrected significant variant in HA, rs1514175 (*TNNI3K*), which showed a comparatively stronger association in smokers vs. nonsmokers. Sex-stratified analyses revealed that, in general, smoking had a greater estimated effect on %MBMI in adolescent females. In particular, EA female smokers who carry the *MC4R* obesity susceptibility allele had a %MBMI that was 5.48 % higher than nonsmokers that carry the allele (*p* = 8.4E–06).

Our results are consistent with previous literature, in that not all obesity susceptibility loci showed a greater estimated positive effect in smokers (data not shown). Among EA, 20 of 33 BMI loci (61 %) had a larger estimated effect in smokers versus nonsmokers, while 6 of 12 (50 %) and 12 of 22 BMI loci (55 %) had a larger estimated effect in smokers versus nonsmokers among AA and HA, respectively. In addition, the interaction effects we observed were generally more pronounced in women than in men. Previous analysis of 14 established BMI loci in EA and AA adults found no significant interaction (*p* < 0.05) between BMI SNPs and smoking [33]. However, the authors noted a 3x increase in the estimated effect of the *FTO* (rs9939609) risk allele in EA female smokers, as well as a suggestive stronger estimated effect of the *TMEM18* risk allele in AA female former/never smokers. No differential effects were reported for men. In our analysis, EA female smokers had a 1.22x increase in the estimated effect of the *FTO* risk allele, while EA male smokers had a 1.17 increased estimated effect of the *FTO* risk allele, compared to nonsmokers. We did not examine the effect of *TMEM18* on BMI in AA, as that SNP did not generalize in the recent AA GWAS.

In our study, HA adolescent smokers carrying the obesity risk variant rs1514174 (near *TNNI3K*) were 8.46 %MBMI units larger than their non-smoking peers (*p* = 5.9E–05). The association of *TNNI3K* with obesity has been replicated in both in EA children [38, 41] and HA women [42]. *TNNI3K* has been associated with increased intake of fats and sugary foods in overweight or obese adults with Type 2 diabetes [43], and has been nominally associated (*p* < 0.05) with emotional and uncontrolled eating, suggesting a potential mechanism for influencing obesity [30]. In mouse models, *TNNI3K* expression has been linked to cardiac function and cardiac oxidative stress following myocardial infarction [44, 45]. Both smoking and obesity increase systemic oxidative stress [46] and risk of cardiovascular disease (CVD), and the influence of *TNNI3K* on cardiac function suggests a possible biological pathway for this interaction.

Two loci showed nominally significant effects for interaction in EA adolescents, *POC5* and *MC4R.* EA adolescents carrying the obesity risk variant rs2112347 (near *POC5*) were 1.98 %MBMI units larger than EA adolescent nonsmokers. Though the association between rs2112347 and BMI has been replicated [41, 47, 48], the biological mechanism through which rs2112347 influences obesity risk in not known [49]. This variant does lie, however, within 500 kb of *HMGCR*, a gene involved in lipid metabolism. Cigarette smoking increases dyslipidemia by inducing lipolysis in adipose tissue [50, 51], offering a promising avenue for future studies.

Finally, rs571312 near *MC4R* demonstrates the strongest influence on %MBMI in EA female smokers [β = 5.48 (3.06, 7.88), *p* = 8.4E-06], compared to EA female nonsmokers [β 1.11 (−0.79, 3.01), *p* = 0.253], EA male nonsmokers [β 2.05 (0.07, 4.03), *p* = 0.042], and EA male smokers [β 0.87 (−1.58, 3.32), *p* = 0.489] (Table 2, Fig. 2). Variants in *MC4R* are associated with monogenic obesity and show differential effects on BMI by sex and age, with a greater influence on adolescent females [22, 52]. *MC4R* is primarily expressed in the central nervous system [53], and plays a pivotal role in the leptin-melanocortin pathway regulating appetite, energy balance, and stress response [54]. Variants in and near *MC4R* have been linked to metabolic syndrome [55, 56], percent body fat [57, 58], eating behavior [59], higher fat intake [60], and lower energy expenditure [61, 62]. In animal and some human models, variants near *MC4R* have been shown to disproportionally affect adiposity in females [63–69]. While nicotine has been implicated in animal models as having a hypophagic effect on the leptin-melanocortin pathway influencing feeding behavior [37, 70], other research has shown a 2.9 fold increased risk of metabolic syndrome among smokers who carry a risk variant at a SNP (rs17782313) in high linkage disequilibrium (LD) with our *MC4R* SNP (rs571312, *R*^*2*^ = 0.955) [69]. Rs17782313 has also been associated with a gender and temporal-specific effect on BMI, as well as smoking behavior [72]. Our results suggest *MC4R* obesity risk variants might mitigate the appetite suppressant effect of nicotine in adolescent female smokers.

Add Health represents a unique sample during a sensitive developmental period, when risky health behaviors are being established. Add Health is a nationally representative sample of US adolescents who are being followed into adulthood. As such, our results can be considered generalizable to American adolescents entering adulthood in the late 1990s-early 2000s, but likely are not generalizable to adolescents at other time periods or in other countries. While we are fortunate to have measured heights and weights for the majority of our sample, current smoking was self-reported, though the questions used to assess smoking status in Add Health have been validated among adolescents. Our study was also limited by the lack of established BMI loci in all ancestries, particularly HA. We also recognize that we were possibly underpowered to detect effects due to small sample size [73], and that our approach cannot account for SNPs with an interaction effect but no measurable marginal effect on %MBMI. Given our sample size (*N* = 5075) and other model parameters in EA, we have between 47 and 52 % power to detect nominally significant interaction effects as large as those seen for the variants near *POC5* (β 1.98) and *MC4R* (β 2.15). While our power is limited, pointing to the need to replicate our results in larger future studies, our results do suggest potential SNPs for further interrogation of the influence of smoking on BMI, particularly in adolescent females.

## Conclusions

Our study highlights potentially important sex differences in obesity risk by smoking status in adolescents, with those who may be most likely to initiate smoking (i.e., adolescent females), being at greatest risk for poor health outcomes (exacerbating genetic obesity risk). Smoking influences central body fat distribution, and research suggests this effect could be particularly pronounced among women [74]. In addition, smokers have a greater risk of metabolic syndrome [71, 75] and dyslipidemia [76], as well as a much greater risk of mortality, particularly for CVD deaths among obese women under age 65 [3], highlighting the importance of targeting smoking early in adolescence to prevent poorer health in adulthood.

## Methods

### Study sample

The National Longitudinal Study of Adolescent to Adult Health (Add Health) is a nationally-representative, prospective cohort of adolescents from the US school-based population in grades 7 to 12 (12–20 years of age) in 1994–95 (*n* = 20,745) who have been followed into adulthood (Table 2). Add Health selected a systematic random sample of 80 high schools and 52 feeder middle schools, representative of US schools with respect to region, urbanicity, school type and size, and student demographics. Written informed consent was obtained from participants or a parent/guardian if the participant was a minor at the time of recruitment. Respondents were followed through Wave II (1996, *n* = 14,738, age 13–21), Wave III (2001–2002, *n* = 15,197, age 18–26) and most recently Wave IV (2007–2008, *n* = 15,701, age 24–32), when respondents provided written informed consent for participation in genetic studies (*n* = 12,234). Add Health included a core sample plus subsamples of selected groups, including African American students with at least one parent with a college degree, collected under protocols approved by the Institutional Review Board at the University of North Carolina at Chapel Hill covering recruitment at all sites. The survey design and sampling frame have been described previously [77-79].

### Race/ethnicity

Ancestry informative genetic markers were not available, so a self-reported race/ethnicity variable was constructed based on survey responses regarding ancestral background and family relationship status from both participants and their parents at Wave I. We used a three-category classification: non-Hispanic European American (EA), non-Hispanic African American (AA), and Hispanic American (HA). Within HA, we generated additional variables to account for subpopulation (Cuban, Puerto Rican, Central/South American, Mexican, or Other Hispanic), as well as foreign-born status (first generation immigrants versus those born in the US).

### Sibling relatedness

Add Health oversampled related adolescents, resulting in 5524 related Wave I respondents living in 2639 households [80]. Familial relatedness was classified according to participant and parental self-report. Twin zygosity was confirmed by 11 molecular genetic markers [81].

### Genetic characterization

The 40 SNPs genotyped in the current study were identified in published GWAS from the Genetic Investigation of Anthropometric Traits (GIANT) consortium for BMI in EA adults [16, 18, 19, 21]. Genotyping was performed using TaqMan assays and the ABI Prism 7900R Sequence Detection System (Applied Biosystems, Foster City, CA, USA). Primer sequences and TaqMan probes are available upon request. The genotype call rate ranged from 97.8 to 98.2 % and the discordance rate between blind duplicates was 0.3 %. SNPs that failed tests for Hardy-Weinberg Equilibrium (HWE) (*p* < 0.001) within race/ethnicity were excluded (*N* = 1, rs2922763) resulting in 39 SNPs for this analysis, as listed in Additional file 5: Table S5.

### Criteria for generalizability

Across all groups, to the extent possible, generalizability was defined as similar direction of effect as reported in the literature and nominal statistical significance (*p* < 0.05) [24]. These criteria make generalization in the EA subpopulation straightforward, since these associations were defined in EA adults. A recent large AA GWAS [82], however, suggests that some SNPs fail to generalize, either due to limited power or because of linkage disequilibrium differences that fail to capture the signal of the functional variant. We thus excluded 15 SNPs in AA that have not shown evidence for generalization (i.e., SNP effect estimates were directionally inconsistent and evidence for association was *p* > 0.20 in the recent AA GWAS) [82]. Similar results were reported in a recent HA GWAS of postmenopausal women, where only 9 of 32 established BMI loci showed evidence for association. As this analysis was conducted in a limited sample, however, we chose to retain all directionally consistent loci in our HA analysis [42]. In addition, SNPs with insufficient cell size for analysis (*n* < 10 individuals per genotype) were excluded, leaving 33 SNPs in EA, 12 SNPs in AA, and 22 SNPs in HA for the interaction analyses (included SNPs highlighted in **bold** in Additional file 5: Table S5).

### Analytic sample

At Wave IV, 59 % (*n* = 12,234) of Wave I (*n* = 20,745) respondents provided samples, with consent, from which DNA was extracted and genotyped (*n* = 12,066). To be eligible our study, individuals had to have at least 80 % of their 39 SNPs genotyped (*n* = 11,448) and be between the ages of 12 and 21 years at either Wave II or III (*n* = 9129). Among the 9129 eligible adolescents, we excluded: the monozygotic twin with fewer genotyped loci (*n* = 139), individuals of Native American (*n* = 57), Asian (*n* = 436) or unclassified (*n* = 112) race/ethnicity, pregnant (*n* = 110), disabled (*n* = 47), and those missing data for geographic region (*n* = 67), BMI (*n* = 2), or current smoking (*n* = 46). The analytic sample was selected from waves II or III to capture the age range of 12–21 years, and all covariates match the wave at which BMI was measured. Our final analytic sample (*n* = 8113) included 5075 EA, 1744 AA, and 1294 HA.

### Body mass index (BMI)

Weight and height were measured during in-home surveys using standardized procedures. BMI (kg/m^2^) was calculated using measured height and weight assessed at Waves II or III when participants were between the age of 12 and 21 years, with priority for younger age at measurement (Wave II: *n* = 7681), unless the respondent was not seen at Wave II and was still between the ages of 12–21 years at Wave III (*n* = 432). Self-reported heights and weights, which have been previously validated in Add Health, were substituted for those who refused measurement and/or weighed more than the scale capacity (Wave II *n* = 55; Wave III *n* = 24) [83]. Due to changes in weight and height with growth and development, BMI varies by age and sex, which necessitates using age- and sex-specific BMI Z-scores relative to a reference such as the US the CDC/NCHS 2000 growth curves [84]. However, these growth curves do not represent the tails of the distribution well, which is a particular issue in a cohort with considerable upward skew in distribution relative to the CDC/NCHS 2000 healthy reference. A strategy to deal with this is to use percent of the CDC/NCHS 2000 median [85], which also has the benefit of ease in interpretation relative to the Z-score. Accordingly, our outcome for all analyses was the percent of the CDC/NCHS 2000 median BMI (%MBMI).

### Current smoking

Current smoking was based on self-report, which has been previously validated among adolescents [86], and was defined as smoking at least 1 day in the last 30 days. [2, 87, 88] Current smoking status was queried at Waves II (N_current_smokers_ = 2589) and III (N_current_smokers_ = 2377), to match the wave at which BMI was measured. To measure the effect of BMI-related SNPs on current smoking, we performed main effects logistic regression using smoking status as the outcome and SNP as predictor, stratified by ancestry (Additional file 2: Tables S2, Additional file 3: Tables S3 and Additional file 4: Tables S4).

### Statistical analysis

In ancestry-stratified, multivariable interaction (SNPxsmoking) models with %MBMI as the outcome, we controlled for age, sex, geographic region, and self-reported heights and weights using Stata (v13.1, Stata Corp, College Station, Texas). In non-EA populations, we also controlled for oversampling of adolescents from highly-educated African American families (*n* = 355), and Hispanic subpopulation: Cuban (*n* = 193), Puerto Rican (*n* = 223), Central/South American (*n* = 119), Mexican (*n* = 656), and other Hispanic (*n* = 102), as well as an indicator for foreign-born status (*n* = 267). Sample design effects and familial relatedness were accounted for by including separate random effects for school and family. When a nominally significant interaction (*p* < 0.05) was detected, we ran additional interaction models (SNP x smoking status, stratified by sex; and SNP x smoking status x sex), and examined SNP effects in models stratified by smoking status and sex, to facilitate interpretation. To correct for multiple testing, we applied a Bonferroni correction equal to 0.05/number of SNPs tested in each group (0.05/33 = 0.0015 in EA, 0.05/22 = 0.0023 in HA, 0.05/12 = 0.0042 in AA).

### Availability of data and materials

Add Health adheres to the NIH policy on data sharing, but due to the sensitive nature of Add Health data, access is limited and governed by the Add Health data management security plan to ensure respondent confidentiality. For this reason, the distribution of data is limited to a public-use dataset for a subset of respondents, and a restricted-use dataset distributed only to certified researchers committed to maintaining limited access. Add Health is currently in the process of submitting genetic data to dbGaP, which will be made available to researchers meeting both dbGaP and Add Health data use requirements. More information can be found here: http://www.cpc.unc.edu/projects/addhealth.

## Additional files

Additional file 1: Table S1. Two-sample *t*-test of differences in BMI and %MBMI by smoking status, stratified by ancestry and sex. (DOCX 72 kb) Additional file 2: Table S2. Results of SNPxSmoking on %MBMI (Interaction), SNP on %MBMI (Main effects), and SNP on smoking in European American adolescents in Add Health. (DOCX 41 kb) Additional file 3: Table S3. Results of SNPxSmoking on %MBMI (Interaction), SNP on %MBMI (Main effects), and SNP on smoking in African American adolescents in Add Health. (DOCX 35 kb) Additional file 4: Table S4. Results of SNPxSmoking on %MBMI (Interaction), SNP on %MBMI (Main effects), and SNP on smoking in Hispanic American adolescents in Add Health. (DOCX 40 kb) Additional file 5: Table S5. Established BMI loci used in present analysis. (DOCX 31 kb)

## Abbreviations

BMI: Body mass index
%MBMI: Percent median BMI
SNP: Single nucleotide polymorphism
EA: European American
AA: African American
HA: Hispanic American
CVD: Cardiovascular disease
GWAS: Genome Wide Assocation Study
HWE: Hardy Weinberg Equilibrium
