# Supplementary material for: Interaction of smoking and obesity susceptibility loci on adolescent BMI: The National Longitudinal Study of Adolescent to Adult Health
Source: BMC Genet. 2015 Nov 4;16:131. doi: 10.1186/s12863-015-0289-6 (PMC4634717; doi:10.1186/s12863-015-0289-6)
Supplement: Additional file 1: Table S1. — Two-sample t-test of differences in BMI and %MBMI by smoking status, stratified by ancestry and sex. (DOCX 72 kb) [file 12863_2015_289_MOESM1_ESM.docx]

Supplementary Table 1. Two-sample t-test of differences in BMI and %MBMI by smoking status, stratified by ancestry and sex.

| **Ancestry** | **BMI [95% CI]** | | **t** | ***p*** |
| --- | --- | --- | --- | --- |
| *Females* | *Smokers* | *Nonsmokers* |  |  |
| EA | 23.1 [22.8, 23.4] | 22.6 [22.4, 22.9] | -2.43 | 0.015 |
| AA | 26.4 [25.3, 27.4] | 24.5 [24.1, 25.0] | -3.23 | 0.001 |
| HA | 25.0 [24.1, 25.8] | 23.3 [22.8, 23.7] | -3.71 | 2.0E-04 |
| *Males* | *Smokers* | *Nonsmokers* |  |  |
| EA | 23.2 [22.9, 23.5] | 23.3 [23.0, 23.5] | 0.15 | 0.885 |
| AA | 23.8 [23.0, 24.5] | 23.6 [23.2, 24.0] | -0.48 | 0.631 |
| HA | 24.4 [23.6, 25.2] | 24.1 [23.6, 24.5] | -0.73 | 0.464 |
| **Ancestry** | **%MBMI [95% CI]** | | **t** | ***p*** |
| *Females* | *Smokers* | *Nonsmokers* |  |  |
| EA | 111.56 [110.07, 113.06] | 110.33 [109.18, 111.49] | -1.29 | 0.196 |
| AA | 126.74 [121.57, 131.90] | 118.62 [116.59, 120.65] | -3.05 | 0.002 |
| HA | 120.38 [116.28, 124.48] | 112.02 [109.76, 114.28] | -3.71 | 2.0E-04 |
| *Males* | *Smokers* | *Nonsmokers* |  |  |
| EA | 109.06 [107.63, 110.52] | 111.23 [110.02, 112.43] | 2.24 | 0.025 |
| AA | 111.52 [107.94, 115.09] | 112.34 [110.39, 114.29] | 0.39 | 0.694 |
| HA | 114.33 [110.80, 117.87] | 113.67 [111.45, 115.89] | -0.31 | 0.754 |
