# Supplementary material for: Interaction of smoking and obesity susceptibility loci on adolescent BMI: The National Longitudinal Study of Adolescent to Adult Health
Source: BMC Genet. 2015 Nov 4;16:131. doi: 10.1186/s12863-015-0289-6 (PMC4634717; doi:10.1186/s12863-015-0289-6)
Supplement: Additional file 2: Table S2. — Results of SNPxSmoking on %MBMI (Interaction), SNP on %MBMI (Main effects), and SNP on smoking in European American adolescents in Add Health. (DOCX 41 kb) [file 12863_2015_289_MOESM2_ESM.docx]

Supplementary Table 2. Results of SNPxSmoking on %MBMI (Interaction), SNP on %MBMI (Main effects), and SNP on smoking in European American adolescents in Add Health.

| **European Americans** | | **Interaction** | | | **Main effects** | | | **SNP on Smoking** | | |
| --- | --- | --- | --- | --- | --- | --- | --- | --- | --- | --- |
| In/nearest gene | N | beta | 95% CI | p | Beta | 95% CI | p | beta | 95% CI | p |
| *ADCY9* | 5043 |  |  |  | -0.46 | -1.38, 0.46 | 0.33 | 1.77E-02 | -1.90E-03, 0.04 | 0.07 |
| *BDNF* | 5041 | -0.32 | -2.57, 1.93 | 0.78 | 1.24 | 0.12, 2.36 | **0.03** | 8.32E-03 | -0.01, 0.03 | 0.48 |
| *CADM2* | 5044 | -1.11 | -3.42, 1.20 | 0.35 | 1.34 | 0.20, 2.48 | **0.02** | 5.45E-04 | -0.02, 0.02 | 0.96 |
| *ETV5* | 5035 | 0.73 | -1.54, 3.00 | 0.53 | 0.53 | -0.59, 1.65 | 0.36 | 2.62E-02 | 0.01, 0.05 | **0.03** |
| *FAIM2* | 4954 | 0.29 | -1.61, 2.19 | 0.77 | 0.69 | -0.25, 1.63 | 0.15 | -4.05E-03 | -0.02, 0.02 | 0.68 |
| *FANCL* | 5050 | -0.82 | -2.86, 1.22 | 0.43 | 1.34 | 0.32, 2.36 | **0.01** | -9.90E-04 | -0.02, 0.02 | 0.93 |
| *FTO* | 5027 | 0.52 | -1.36, 2.40 | 0.59 | 3.48 | 2.54, 4.42 | **3.76E-13** | -1.39E-02 | -0.03, 0.01 | 0.16 |
| *GNPDA2* | 5035 | -0.84 | -2.72, 1.04 | 0.38 | 1.42 | 0.48, 2.36 | **3.00E-03** | 1.65E-02 | -3.10E-03, 0.04 | 0.09 |
| *GPRC5B* | 5030 | -0.24 | -2.85, 2.37 | 0.86 | 1.07 | -0.22, 2.36 | 0.11 | 3.73E-03 | -0.02, 0.02 | 0.78 |
| *KCTD15* | 5021 | 1.19 | -0.77, 3.15 | 0.23 | 0.68 | -0.30, 1.66 | 0.17 | 1.95E-03 | -0.02, 0.02 | 0.85 |
| *LMX1B* | 5042 | 0.32 | -1.97, 2.61 | 0.78 | 0.54 | -0.60, 1.68 | 0.35 | -1.41E-02 | -0.03, 0.01 | 0.24 |
| *LRP1B* | 5038 | -1.79 | -4.28, 0.70 | 0.16 | 0.59 | -0.64, 1.82 | 0.35 | -4.77E-03 | -0.02, 0.01 | 0.71 |
| *LRRN6C* | 5015 | 1.25 | -0.73, 3.23 | 0.22 | 0.34 | -0.64, 1.32 | 0.5 | -1.07E-02 | -0.03, 0.01 | 0.3 |
| *LZTR2* | 5047 | 0.44 | -1.83, 2.71 | 0.7 | 1.85 | 0.71, 2.99 | **1.35E-03** | -1.73E-03 | -0.02, 0.02 | 0.88 |
| *MAF* | 5032 |  |  |  | -0.35 | -1.27, 0.57 | 0.46 | 3.76E-03 | -0.02, 0.02 | 0.7 |
| *MAP2K5* | 5045 | 0.86 | -1.30, 3.02 | 0.44 | 1.24 | 0.16, 2.32 | **0.02** | 2.39E-04 | -0.02, 0.02 | 0.98 |
| *MC4R* (rs12970134) | 5041 | 1.57 | -0.51, 3.65 | 0.14 | 1.68 | 0.64, 2.72 | **1.46E-03** | -3.22E-03 | -0.02, 0.02 | 0.77 |
| *MC4R* (rs571312) | 5047 | 2.15 | -0.03, 4.33 | **0.05** | 2.31 | 1.23, 3.39 | **2.65E-05** | -4.29E-04 | -0.02, 0.02 | 0.97 |
| *MTCH2* | 5024 | -1.04 | -2.90, 0.82 | 0.28 | 0.97 | 0.05, 1.89 | **0.04** | -3.44E-03 | -0.02, 0.02 | 0.72 |
| *MTIF3* | 5029 |  |  |  | -0.41 | -1.51, 0.69 | 0.46 | 2.17E-02 | 2.10E-03, 0.04 | 0.06 |
| *NCR3/BAT2* | 5007 |  |  |  | -0.27 | -1.17, 0.63 | 0.55 | -1.24E-02 | -0.03, 0.01 | 0.2 |
| *NEGR1* | 5022 | -0.01 | -1.91, 1.89 | 0.99 | 1.03 | 0.09, 1.97 | **0.03** | 2.95E-02 | 0.01, 0.05 | **2.94E-03** |
| *NPC1* | 5013 | -0.49 | -2.37, 1.39 | 0.61 | 0.36 | -0.58, 1.30 | 0.45 | -5.32E-03 | -0.02, 0.01 | 0.59 |
| *NRXN3* | 5060 |  |  |  | -1.12 | -2.26, 0.02 | **0.05** | 9.15E-03 | -0.01, 0.03 | 0.44 |
| *NUDT3* | 5057 | 1.46 | -0.79, 3.71 | 0.2 | 1.26 | 0.14, 2.38 | **0.03** | 2.49E-02 | 0.01, 0.04 | **0.03** |
| *POC5* | 5035 | 1.98 | 0.06, 3.90 | **0.04** | 0.78 | -0.18, 1.74 | 0.11 | -5.62E-03 | -0.03, 0.01 | 0.58 |
| *POMC* | 5022 | -0.25 | -2.11, 1.61 | 0.79 | 1.46 | 0.54, 2.38 | **1.94E-03** | -3.16E-03 | -0.02, 0.02, 0.02 | 0.74 |
| *PRL* | 5037 | -0.72 | -2.60, 1.16 | 0.45 | 0.61 | -0.33, 1.55 | 0.2 | 1.97E-04 | -0.02, 0.02 | 0.98 |
| *PTBP2* | 5038 | 0.03 | -1.83, 1.89 | 0.98 | 1.13 | 0.21, 2.05 | **0.02** | -5.94E-03 | -0.03, 0.01 | 0.54 |
| *QPCTL* | 5050 | -1.19 | -3.56, 1.18 | 0.33 | 0.53 | -0.65, 1.71 | 0.38 | 2.69E-02 | 0.01, 0.05 | **0.03** |
| *RPL27A* | 5031 |  |  |  | -0.51 | -1.41, 0.39 | 0.27 | -7.28E-03 | -0.03, 0.01 | 0.45 |
| *SEC16B* | 5055 | 0.13 | -2.10, 2.36 | 0.91 | 1.68 | 0.56, 2.80 | **3.23E-03** | 7.42E-03 | -0.01, 0.03 | 0.53 |
| *SH2B1* | 5049 | 0.33 | -1.53, 2.19 | 0.73 | 0.42 | -0.50, 1.34 | 0.37 | 1.55E-02 | -4.10E-03, 0.04 | 0.11 |
| *SH2B1/*  *APOB48* | 5035 | 0.04 | -1.82, 1.90 | 0.96 | 0.41 | -0.51, 1.33 | 0.38 | 1.54E-02 | -4.20E-03, 0.04 | 0.11 |
| *SLC39A8* | 5053 | 0.26 | -3.09, 3.61 | 0.88 | 0.85 | -0.84, 2.54 | 0.32 | 7.42E-03 | -0.03, 0.05 | 0.67 |
| *TFAP2B* | 5055 | -2.17 | -4.58, 0.24 | 0.08 | 2.6 | 1.40, 3.80 | **1.89E-05** | -7.82E-03 | -0.03, 0.01 | 0.53 |
| *TMEM160/ ZC3H4* | 5032 | -0.13 | -2.09, 1.83 | 0.9 | 0.51 | -0.45, 1.47 | 0.3 | 3.22E-03 | -0.02, 0.02 | 0.75 |
| *TMEM18* | 5053 | -0.45 | -2.82, 1.92 | 0.71 | 2.99 | 1.79, 4.19 | **8.87E-07** | -9.98E-03 | -0.03, 0.01 | 0.43 |
| *TNNI3K* | 5048 | 0.21 | -1.63, 2.05 | 0.82 | 1.03 | 0.11, 1.95 | **0.03** | -7.27E-03 | -0.03, 0.01 | 0.45 |

**Bold** highlights nominally significant associations (*p* ≤ 0.05). Interaction tests were not performed for SNPs that did not show directionally consistent main effects. %MBMI = Percent of the CDC/NCHS 2000 median BMI.
