# Supplementary material for: Interaction of smoking and obesity susceptibility loci on adolescent BMI: The National Longitudinal Study of Adolescent to Adult Health
Source: BMC Genet. 2015 Nov 4;16:131. doi: 10.1186/s12863-015-0289-6 (PMC4634717; doi:10.1186/s12863-015-0289-6)
Supplement: Additional file 3: Table S3. — Results of SNPxSmoking on %MBMI (Interaction), SNP on %MBMI (Main effects), and SNP on smoking in African American adolescents in Add Health. (DOCX 35 kb) [file 12863_2015_289_MOESM3_ESM.docx]

Supplementary Table 3. Results of SNPxSmoking on %MBMI (Interaction), SNP on %MBMI (Main effects), and SNP on smoking in African American adolescents in Add Health.

| **African Americans** | | **Interaction** | | | **Main effects** | | | **SNP on Smoking** | | |
| --- | --- | --- | --- | --- | --- | --- | --- | --- | --- | --- |
| In/nearest gene | N | beta | 95% CI | p | beta | 95% CI | p | beta | 95% CI | p |
| *ADCY9* | 1726 | -0.01 | -5.62, 5.61 | 1.00 | 1.18 | -0.99, 3.35 | 0.29 | 1.45E-02 | -0.02, 0.04 | 0.35 |
| *ETV5* | 1733 | 0.75 | -3.97, 5.47 | 0.76 | 2.32 | 0.48, 4.16 | 0.01 | 7.85E-03 | -0.02, 0.03 | 0.55 |
| *FTO* | 1734 |  |  |  | -0.31 | -2.13, 1.50 | 0.73 | -5.35E-03 | -0.03, 0.02 | 0.68 |
| *GNPDA2* | 1731 | -2.38 | -8.03, 3.27 | 0.41 | 3.79 | 1.58, 5.99 | **7.80E-04** | -1.36E-03 | -0.03, 0.03 | 0.93 |
| *LMX1B* | 1733 |  |  |  | -0.52 | -2.52, 1.47 | 0.61 | 1.62E-03 | -0.03, 0.03 | 0.91 |
| *LRRN6C* | 1726 | -3.73 | -9.50, 2.05 | 0.21 | 2.26 | -0.12, 4.63 | 0.06 | 2.56E-02 | -0.01, 0.06 | 0.13 |
| *LZTR2* | 1730 | -1.48 | -6.89, 3.93 | 0.59 | 3.72 | 1.59, 5.86 | **6.34E-04** | 2.91E-02 | 0.00, 0.06 | 0.06 |
| *MAF* | 1733 | 4.86 | -0.29, 10.01 | 0.06 | 0.16 | -1.83, 2.16 | 0.87 | 6.60E-03 | -0.02, 0.03 | 0.64 |
| *MAP2K5* | 1736 | 1.64 | -3.20, 6.47 | 0.51 | 2.28 | 0.40, 4.16 | 0.02 | -8.08E-03 | -0.03, 0.02 | 0.55 |
| *MTCH2* | 1733 | -0.19 | -5.46. 5.08 | 0.94 | 0.72 | -1.36, 2.81 | 0.50 | 1.96E-02 | -0.01, 0.05 | 0.18 |
| *NCR3_BAT2* | 1722 | 2.43 | -2.63, 7.49 | 0.35 | 0.06 | -1.88, 2.00 | 0.95 | 4.95E-03 | -0.02, 0.03 | 0.72 |
| *NRXN3* | 1735 |  |  |  | -0.46 | -2.34, 1.42 | 0.63 | -2.53E-02 | -0.05, 0.00 | 0.06 |
| *NUDT3* | 1732 |  |  |  | -0.48 | -2.32, 1.36 | 0.61 | 6.76E-04 | -0.03, 0.03 | 0.96 |
| *PRL* | 1730 |  |  |  | -0.43 | -2.39, 1.53 | 0.67 | 2.81E-02 | 0.00, 0.06 | **0.04** |
| *SEC16B* | 1738 | 0.77 | -4.26, 5.81 | 0.76 | 4.42 | 2.41, 6.44 | **1.67E-05** | 2.99E-02 | 0.00, 0.06 | **0.04** |
| *SH2B1* | 1726 | 0.19 | -4.95, 5.34 | 0.94 | 0.92 | -1.14, 2.98 | 0.38 | 9.64E-03 | -0.02, 0.04 | 0.51 |
| *TMEM160* | 1726 | -1.66 | -7.36, 4.04 | 0.57 | 1.30 | -1.00, 3.59 | 0.27 | 2.04E-02 | -0.01, 0.05 | 0.21 |

**Bold** highlights nominally significant associations (*p* ≤ 0.05). Interaction tests were not performed for SNPs that did not show directionally consistent main effects. %MBMI = Percent of the CDC/NCHS 2000 median BMI.
