# Supplementary material for: Interaction of smoking and obesity susceptibility loci on adolescent BMI: The National Longitudinal Study of Adolescent to Adult Health
Source: BMC Genet. 2015 Nov 4;16:131. doi: 10.1186/s12863-015-0289-6 (PMC4634717; doi:10.1186/s12863-015-0289-6)
Supplement: Additional file 4: Table S4. — Results of SNPxSmoking on %MBMI (Interaction), SNP on %MBMI (Main effects), and SNP on smoking in Hispanic American adolescents in Add Health. (DOCX 40 kb) [file 12863_2015_289_MOESM4_ESM.docx]

Supplementary Table 4. Results of SNPxSmoking on %MBMI (Interaction), SNP on %MBMI (Main effects), and SNP on smoking in Hispanic American adolescents in Add Health.

| **Hispanic Americans** | | | **Interaction** | | | **Main effects** | | | **SNP on smoking** | | |
| --- | --- | --- | --- | --- | --- | --- | --- | --- | --- | --- | --- |
| In/nearest gene | N | beta | | 95% CI | p | beta | 95% CI | p | beta | 95% CI | p |
| *ADCY9* | 1286 |  | |  |  | -1.43 | -3.44, 0.59 | 0.17 | -2.51E-02 | -0.06, 0.01 | 0.17 |
| *BDNF* | 1282 | -0.57 | | -5.83, 4.68 | 0.83 | 2.51 | 0.02, 4.99 | **0.05** | -4.24E-02 | -0.09, 0.00 | 0.06 |
| *ETV5* | 1287 |  | |  |  | -0.89 | -3.38, 1.61 | 0.49 | 1.03E-02 | -0.03, 0.05 | 0.65 |
| *FAIM2* | 1265 | -1.26 | | -6.19, 3.67 | 0.62 | 0.16 | -2.08, 2.39 | 0.89 | -3.17E-03 | -0.04, 0.04 | 0.88 |
| *FANCL* | 1289 |  | |  |  | -1.42 | -3.95, 1.11 | 0.27 | 2.25E-02 | -0.02, 0.07 | 0.33 |
| *FTO* | 1286 | -1.27 | | -5.81, 3.27 | 0.58 | 1.68 | -0.39, 3.76 | 0.11 | -1.12E-02 | -0.05, 0.03 | 0.55 |
| *GNPDA2* | 1288 | -2.13 | | -6.52, 2.26 | 0.34 | 1.46 | -0.57, 3.49 | 0.16 | -1.49E-02 | -0.05, 0.02 | 0.41 |
| *KCTD15* | 1279 | 0.90 | | -3.54, 5.33 | 0.69 | 0.73 | -1.32, 2.77 | 0.49 | -2.93E-02 | -0.07, 0.01 | 0.11 |
| *LMX1B* | 1288 |  | |  |  | -0.38 | -2.39, 1.63 | 0.71 | -2.15E-02 | -0.06, 0.01 | 0.24 |
| *LRRN6C* | 1274 | 2.14 | | -3.06, 7.34 | 0.42 | 2.08 | -0.23, 4.38 | 0.08 | -2.82E-02 | -0.07, 0.01 | 0.18 |
| *LZTR2* | 1282 | -2.75 | | -8.29, 2.79 | 0.33 | 2.48 | 0.01, 4.94 | **0.05** | -2.22E-02 | -0.07, 0.02 | 0.32 |
| *MAF* | 1280 | 0.65 | | -3.71, 5.01 | 0.77 | 1.45 | -0.56, 3.46 | 0.16 | 9.43E-03 | -0.03, 0.04 | 0.60 |
| *MAP2K5* | 1285 |  | |  |  | -0.91 | -2.89, 1.07 | 0.37 | 8.34E-03 | -0.03, 0.04 | 0.64 |
| *MTCH2* | 1284 | -4.28 | | -8.61, 0.05 | 0.06 | 1.23 | -0.75, 3.20 | 0.22 | 7.62E-03 | -0.03, 0.04 | 0.67 |
| *MTIF3* | 1287 | 2.19 | | -2.97, 7.36 | 0.41 | 1.37 | -1.09, 3.83 | 0.27 | 4.34E-02 | 0.00, 0.09 | **0.05** |
| *NCR3/BAT2* | 1278 | -0.85 | | -5.11, 3.41 | 0.70 | 0.88 | -1.04, 2.81 | 0.37 | -2.05E-02 | -0.05, 0.01 | 0.24 |
| *NEGR1* | 1285 | 1.05 | | -3.57, 5.66 | 0.66 | 1.04 | -1.08, 3.17 | 0.34 | -3.38E-02 | -0.07, 0.00 | 0.08 |
| *NPC1* | 1287 | -3.13 | | -8.03, 1.77 | 0.21 | 1.16 | -1.05, 3.36 | 0.31 | -2.48E-02 | -0.06, 0.01 | 0.21 |
| *NRXN3* | 1293 |  | |  |  | -0.55 | -2.91, 1.81 | 0.65 | -3.84E-02 | -0.08, 0.00 | 0.07 |
| *NUDT3* | 1280 |  | |  |  | -0.49 | -2.53, 1.54 | 0.63 | 2.16E-02 | -0.01, 0.06 | 0.24 |
| *POC5* | 1286 | 0.60 | | -3.94, 5.13 | 0.80 | 1.47 | -0.60, 3.54 | 0.16 | 1.75E-02 | -0.02, 0.05 | 0.35 |
| *POMC* | 1278 |  | |  |  | -1.85 | -3.81, 0.11 | 0.06 | 2.09E-03 | -0.03, 0.04 | 0.91 |
| *PRL* | 1286 | 0.83 | | -3.48, 5.13 | 0.71 | 0.10 | -1.90, 2.10 | 0.92 | 1.57E-02 | -0.02, 0.05 | 0.38 |
| *PTBP2* | 1280 | 1.10 | | -3.22, 5.42 | 0.62 | 0.93 | -1.07, 2.93 | 0.36 | 1.87E-02 | -0.02, 0.05 | 0.30 |
| *RPL27A* | 1276 | -2.17 | | -6.44, 2.10 | 0.32 | 0.09 | -1.85, 2.04 | 0.92 | 7.08E-03 | -0.03, 0.04 | 0.69 |
| *SEC16B* | 1285 | -1.79 | | -7.28, 3.70 | 0.52 | 2.26 | -0.22, 4.73 | 0.07 | -1.98E-02 | -0.06, 0.02 | 0.38 |
| *SH2B1* | 1288 | 0.44 | | -3.87, 4.75 | 0.84 | 1.12 | -0.87, 3.11 | 0.27 | 2.06E-02 | -0.01, 0.06 | 0.25 |
| *SH2B1/APOB48* | 1286 | 1.17 | | -3.20, 5.53 | 0.60 | 0.78 | -1.23, 2.79 | 0.45 | 2.58E-02 | -0.01, 0.06 | 0.16 |
| *TFAP2B* | 1286 | 1.77 | | -2.84, 6.38 | 0.45 | 2.15 | 0.01, 4.30 | **0.05** | 2.10E-02 | -0.02, 0.06 | 0.28 |
| *TMEM160* | 1284 |  | |  |  | -0.55 | -2.56, 1.45 | 0.59 | 1.52E-02 | -0.02, 0.05 | 0.40 |
| *TNNI3K* | 1285 | 8.46 | | 4.33, 12.59 | **5.87E-05** | 0.64 | -1.31, 2.59 | 0.52 | -1.05E-02 | -0.04, 0.02 | 0.55 |

**Bold** highlights nominally significant associations (*p* ≤ 0.05). Interaction tests were not performed for SNPs that did not show directionally consistent main effects. %MBMI = Percent of the CDC/NCHS 2000 median BMI.
