# Supplementary material for: Interaction of smoking and obesity susceptibility loci on adolescent BMI: The National Longitudinal Study of Adolescent to Adult Health
Source: BMC Genet. 2015 Nov 4;16:131. doi: 10.1186/s12863-015-0289-6 (PMC4634717; doi:10.1186/s12863-015-0289-6)
Supplement: Additional file 5: Table S5. — Established BMI loci used in present analysis. (DOCX 31 kb) [file 12863_2015_289_MOESM5_ESM.docx]

Supplementary Table 5. Established BMI loci used in present analysis.

| In or Nearest Gene | SNP | Risk Allele | EA Freq | AA Freq | HA Freq |
| --- | --- | --- | --- | --- | --- |
| *ADCY9* | rs2444217 | A | 0.57 | **0.76** | 0.43 |
| *BDNF* | rs10767664 | A | **0.79** | 0.93 | **0.81** |
| *CADM2* | rs13078807 | G | **0.20** | 0.05 | 0.15 |
| *ETV5* | rs7647305 | C | **0.79** | **0.60** | 0.81 |
| *FAIM2* | rs7138803 | A | **0.38** | 0.17 | **0.27** |
| *FANCL* | rs887912 | T | **0.28** | 0.10 | 0.19 |
| *FTO* | rs9939609 | A | **0.39** | 0.47 | **0.33** |
| *GNPDA2* | rs10938397 | G | **0.43** | **0.24** | **0.37** |
| *GPRC5B* | rs12444979 | C | **0.86** | 0.91 | 0.91 |
| *KCTD15* | rs29941 | G | **0.68** | 0.82 | **0.64** |
| *LMX1B* | rs867559 | G | **0.19** | 0.30 | 0.33 |
| *LRP1B* | rs2890652 | C | **0.16** | 0.30 | 0.13 |
| *LRRN6C* | rs10968576 | G | **0.31** | **0.17** | **0.24** |
| *LZTR2* | rs543874 | G | **0.20** | **0.24** | **0.19** |
| *MAF* | rs1424233 | T | 0.48 | **0.68** | **0.63** |
| *MAP2K5* | rs2241423 | G | **0.77** | **0.62** | 0.58 |
| *MC4R* | rs571312 | A | **0.23** | 0.35 | 0.16 |
| *MC4R* | rs12970134 | A | **0.26** | 0.15 | 0.17 |
| *MTCH2* | rs3817334 | T | **0.40** | **0.26** | **0.39** |
| *MTIF3* | rs4771122 | G | 0.22 | 0.22 | **0.20** |
| *NCR3/BAT2* | rs1077393 | G | 0.49 | **0.35** | **0.49** |
| *NEGR1* | rs2568958 | A | **0.63** | 0.55 | **0.69** |
| *NPC1* | rs1805081 | G | **0.49** | 0.92 | **0.73** |
| *NRXN3* | rs10146997 | G | 0.79 | 0.64 | 0.79 |
| *NUDT3* | rs206936 | G | **0.21** | 0.54 | 0.40 |
| *POC5* | rs2112347 | T | **0.63** | 0.51 | **0.63** |
| *PRL* | rs4712652 | G | **0.42** | 0.29 | **0.38** |
| *PTBP2* | rs1555543 | C | **0.59** | 0.43 | **0.57** |
| *POMC* | rs713586 | C | **0.48** | 0.84 | 0.43 |
| *QPCTL* | rs2287019 | C | **0.82** | 0.88 | 0.87 |
| *RPL27A* | rs4929949 | C | 0.51 | 0.40 | **0.49** |
| *SEC16B* | rs10913469 | C | **0.20** | **0.30** | **0.19** |
| *SH2B1* | rs4788102 | A | **0.39** | **0.28** | **0.40** |
| *SH2B1/APOB48* | rs7359397 | T | **0.39** | 0.08 | **0.38** |
| *SLC39A8* | rs13107325 | T | **0.08** | 0.02 | 0.04 |
| *TFAP2B* | rs987237 | G | **0.18** | 0.10 | **0.27** |
| *TMEM160/ZC3H4* | rs3810291 | A | **0.67** | **0.21** | 0.56 |
| *TMEM18* | rs6548238 | C | **0.83** | 0.89 | 0.87 |
| *TNNI3K* | rs1514175 | A | **0.44** | 0.67 | **0.53** |

**Bold** risk allele frequencies indicate SNPs which were tested in a particular ancestry group. SNPs were excluded from interaction analyses for lack of generalizability in AA and HA, or directional inconsistency with published GWAS (EA and AA).
